# Acoustic Emission Mechanisms and Fracture Mechanisms in Reinforced Concrete Beams Under Cyclic Loading and Unloading

**DOI:** 10.3390/ma19030521

**Published:** 2026-01-28

**Authors:** Aiping Yu, Tianjiao Miao, Tao Liu, Yuhan Yang, Zhehan Chen

**Affiliations:** 1 School of Civil Engineering, Guilin University of Technology, Guilin 541000, China; apyu@glut.edu.cn (A.Y.); 1020230752@glut.edu.cn (T.M.); 2120230888@glut.edu.cn (T.L.); 2120240949@glut.edu.cn (Y.Y.); 2 School of Civil Engineering, Zhengzhou University, Zhengzhou 450001, China

**Keywords:** acoustic emission, digital image correlation, reinforced concrete beams, fracture mechanisms, damage model

## Abstract

**Highlights:**

**What are the main findings?**

- The correlation between mesoscopic fracture mechanisms and acoustic emission mechanisms in reinforced concrete beams under cyclic loading is revealed by integrating acoustic emission and digital image correlation techniques.
- Acoustic emission signals with distinct spectral characteristics are excited by mesoscopic fracture mechanisms corresponding to different damage stages.

**What are the implications of the main findings?**

- Compared to macro-mechanical indicators such as fracture energy, the acoustic emission parameter Felicity Ratio enables earlier quantification of irreversible damage accumulation.
- An FR-Freq damage model incorporating both time-domain and frequency-domain acoustic emission features is proposed, achieving damage stage identification with an accuracy of 88.89%.

**Abstract:**

This study aims to elucidate the deterministic correlation between the microscopic fracture mechanisms and the multi-domain characteristics of acoustic emission in reinforced concrete beams under cyclic loading. Cyclic incremental tests were designed and conducted, with synchronized application of digital image correlation and AE techniques to capture the entire damage evolution process and corresponding signal responses throughout. The findings reveal that the damage stage division based on mechanical responses is consistent with that based on AE responses. Damage accumulation and irreversible processes can be clearly characterized by AE activity, and the systematic decrease in the Felicity ratio quantitatively verifies the irreversible accumulation of damage. Under cyclic loading, different microscopic fracture mechanisms generate AE frequency-domain signatures with statistically significant differences. A damage identification model integrating the Felicity ratio and multi-band energy features was developed, achieving an accuracy of 88.89% in identifying macroscopic damage stages. This research quantitatively confirms the effectiveness of AE characteristics as reliable identifiers of microscopic fracture mechanisms, providing a new basis for advancing structural health monitoring technologies grounded in fracture mechanism recognition.

## 1. Introduction

Reinforced concrete (RC) beams are inevitably subjected to cyclic loading during their long-term service, and the progressive accumulation of internal damage is the fundamental cause of component performance degradation and even ultimate failure [1]. To capture the evolution of internal damage in real time, acoustic emission (AE) technology, as a dynamic and non-destructive monitoring method [2], is increasingly widely used in the study of fracture processes in concrete structures [3]. Digital image correlation (DIC) enables real-time detection of the surface strain field of the tested structure to capture the damage evolution process. Combining DIC with AE can reduce the assumptions in damage identification using AE [4,5], while the advantage of AE in detecting internal damage effectively compensates for the limitations of DIC.

Existing research, through the integration of multi-parameter acoustic emission monitoring, has made significant progress in areas such as damage localization and qualitative differentiation of failure modes [6,7]. However, most studies focus their analysis on AE activity during the loading phase [8]. There is a lack of systematic investigation into AE behavior during the holding and unloading phases of cyclic loading and the physical significance it carries, failing to fully exploit the complete damage information contained within the entire stress cycle. While parameters such as amplitude, rise time, and average frequency of AE signals can be analyzed to further distinguish different failure modes [9,10], there is an absence of a framework capable of synergistically interpreting multi-dimensional features across the time, frequency, and time-frequency domains and quantitatively correlating them with different stages of the fracture process. These factors, to some extent, constrain the development of damage assessment based on monitoring data towards early warning criteria with clear physical meaning and broad applicability. In reinforced concrete beams, due to the bond interaction between steel reinforcement and concrete, the AE signals generated during their fracture process are more abundant and complex compared to those in plain concrete [11]. AE time parameters such as the RA value and AF value are employed to identify the proportion of tensile cracks versus shear cracks [12,13,14].

Despite the significant potential demonstrated by acoustic emission technology in concrete fracture monitoring, its application to studying the fracture mechanisms of reinforced concrete beams under cyclic loading still faces several challenges and gaps. Existing research has focused more on fracture behavior under monotonic loading or after specific pre-damage [15,16,17,18], while systematic studies on the characteristics of AE signals excited at different stages throughout the complete, progressive evolution of cracks under cyclic incremental loading are still lacking. Previous studies have attempted to identify critical states using sudden changes in the AE hit rate [19,20] or a drop in the b-value [21,22]. However, the robustness and general applicability of these indicators require further verification within the complex context of damage accumulation under cyclic incremental loading. Traditional fracture-based research has primarily concentrated on crack causes, width calculation, and their impact on structural durability [23,24,25,26]. Based on bond-slip theory, scholars have developed various crack width calculation models [27,28,29], and corresponding limit standards have been established in national codes. Recent research has begun to introduce probabilistic and statistical methods [30,31] to describe this process more accurately, constructing the post-cracking steel stress distribution function by analogy with the normal distribution probability density function, thereby establishing stochastic models for crack spacing and width [32,33]. The quantitative relationship between crack width limits and structural durability has also been thoroughly explored [34], with studies indicating the need to set differentiated limits based on the type of environmental erosion and the thickness of the concrete cover [35,36]. While these achievements have significantly deepened the understanding of component cracking performance under static or monotonic loading, limitations remain when addressing the more common cyclic and repeated loading encountered in practical engineering.

This study aims to move beyond the limitation of phenomenological correlation, and is committed to establishing a deterministic mechanistic link between the full-process damage evolution under cyclic loading and the multi-domain characteristics of AE. Cyclic incremental tests were designed, and AE and DIC techniques were employed to achieve identification of the entire damage and failure process. For the first time, the quantitative mapping relationship between the dominant microscopic fracture mechanisms and the AE frequency-domain mechanisms they excite, at different loading stages and damage levels, was systematically revealed. Furthermore, based on the clarified fracture mechanisms, features such as the Felicity ratio and the energy proportion of key frequency bands were selected to construct an FR-Freq damage model that intrinsically links fracture mechanisms with AE mechanisms. This research deepens the understanding of damage accumulation and fracture mechanisms in reinforced concrete under cyclic loading, confirms the potential of AE mechanisms as a reliable means for damage identification, and provides a novel method and theoretical basis for developing structural health monitoring techniques based on fracture mechanisms.

## 2. Experiment and Methods

### 2.1. Sample Preparation

**Figure 1:**
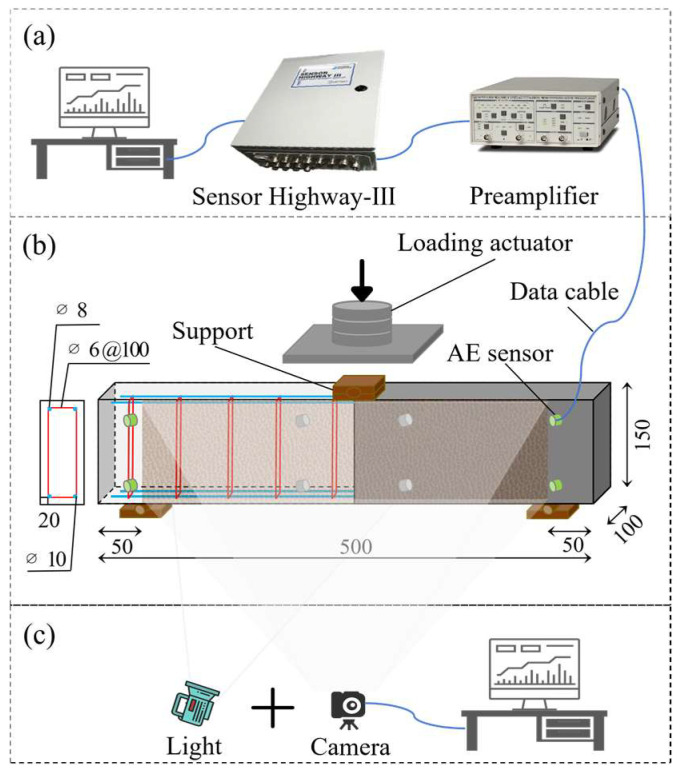
Test system: (**a**) AE detection system, (**b**) loading system, and (**c**) DIC detection system.

The dimensions of the reinforced concrete beam are designed as shown in Figure 1b. The beam has a length of 500 mm, a width of 100 mm, and a depth of 150 mm, with an effective span of 400 mm. The test employed ordinary concrete with a strength grade of C30. The mix proportion was designed in accordance with the Chinese standard [37] and is listed in Table 1. Tap water was used. The cement was ordinary Portland cement (P.O 42.5) produced by Guilin Xing’an Conch Cement Co., Ltd. (Guilin, China), and its properties comply with the Chinese standard [38]. The water-to-binder ratio was 0.5. Fine aggregate consisted of natural river sand with a fineness modulus of 2.83. Its apparent density and bulk density were 2630 kg/m^3^ and 1530 kg/m^3^, respectively, conforming to the Chinese standard [39]. Coarse aggregate was crushed stone with a continuous gradation of 11–16 mm, having an apparent density of 2650 kg/m^3^ and a bulk density of 1460 kg/m^3^. The sand ratio was 0.38. All specimens were cast using the same batch of materials in a single pour and cured under standard conditions for 28 days.

For the compressive strength, cube specimens (150 mm × 150 mm × 150 mm) were prepared simultaneously and tested after standard curing. The average compressive strength of the concrete was 34.5 MPa. Additionally, the average splitting tensile strength of the concrete, measured through standard tests, was 3.8 MPa. Based on the Chinese Code [40], the specimen’s cross-sectional dimensions, and the reinforcement details, the theoretical ultimate flexural capacity of the reinforced concrete beam was calculated to be approximately 110 kN. This value was set as the target loading level for the test and aligned well with the final experimental peak load. All RC beams were reinforced with two 8 mm diameter hanger bars on the compression side and two 10 mm diameter longitudinal bars on the tension side. Stirrups with a diameter of 6 mm were uniformly spaced at 100 mm. All steel bars were of grade HRB 400. The properties of the reinforcing steel materials are shown in Table 2. After curing, a layer of white paint was sprayed on the surface of the RC beams. After natural drying, speckle patterns were applied.

### 2.2. Test System

The experiment consisted of an acoustic emission (AE) detection system, a loading system, and a DIC detection system, as shown in Figure 1. Figure 1a shows the AE detection system, which was responsible for the acquisition, sharpening, and visualization of AE signals. The acquisition parameters were set as follows: preamplifier gain of 26 dB, threshold of 38 dB, and sampling rate of 1 MSPS. The AE equipment included a 32-channel acoustic emission system (Sensor Highway III) from Physical Acoustics Corporation (West Windsor Township, NJ, USA), using PK15I sensors with a resonant frequency of 151 kHz; integrated preamplifier narrowband resonant sensors were used. Figure 1b shows the loading system. The main part of this system was an electro-hydraulic servo universal testing machine. The lower supports were placed 50 mm from each end of the beam. A three-point bending setup was used, with the RC beam configured as a simply supported beam.

Figure 1c shows the DIC detection system. The DIC system employed an MVL-KF2528M-12MP lens produced by Hangzhou Hikrobot Technology Co., Ltd. (Hangzhou, China), featuring a 1.1-inch target size, 12-megapixel effective resolution, and a 25 mm focal length, coupled with a high-resolution sensor. Image acquisition resolution was 5472 × 3648. Subsequent processing was performed using GOM Correlate 2019 software. The camera setup is illustrated in Figure 1. The green sensor array on the front face defines the acquisition area, which was covered with a speckle pattern. Deformation on the beam surface was observed by tracking the positional changes of the same speckle points in images captured by the cameras at different times. To better monitor crack development along the entire span of the RC beam, the speckle pattern covered the full length of the beam. The brown pattern on the beam in the figure represents the speckle area, which measured 400 × 150 mm^2^.

**Figure 2:**
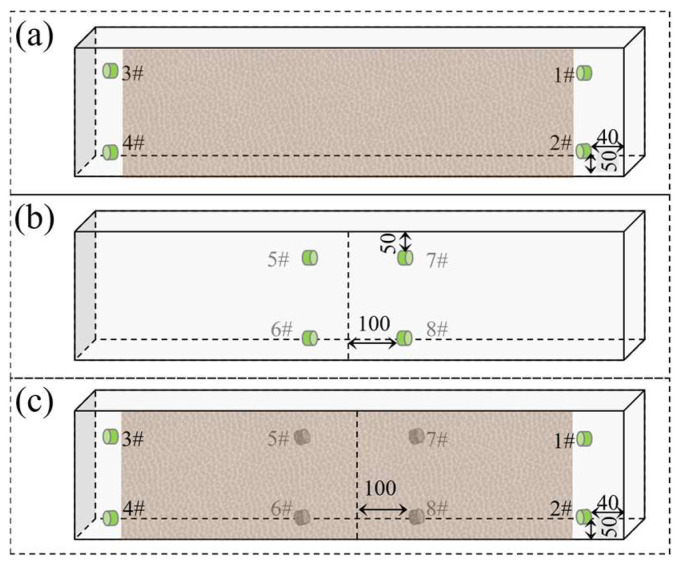
Sensor layout: (**a**) front (speckled), (**b**) back, and (**c**) entirety.

Considering the attenuation and dispersion effects of elastic waves during propagation, sensors were arranged at both ends of potential shear cracks, with two sensors placed on the front and back, respectively, as shown in Figure 2. To maintain low detection costs, sensors were symmetrically arranged to capture as many valid AE signals as possible. Sensors 1#, 3#, 5#, and 7# are on the same horizontal plane, 50 mm from the top surface of the beam. The other four sensors are also on a common horizontal plane, 50 mm from the bottom surface of the beam. The four sensors on the front face are placed close to the speckle pattern, 40 mm from each end of the beam. The sensors on the back face are located 100 mm from the beam’s center line. The beam was roughly divided into three segments for monitoring, with a symmetrical left-right layout and full coverage on both front and back faces.

### 2.3. Testing Procedure

**Figure 3:**
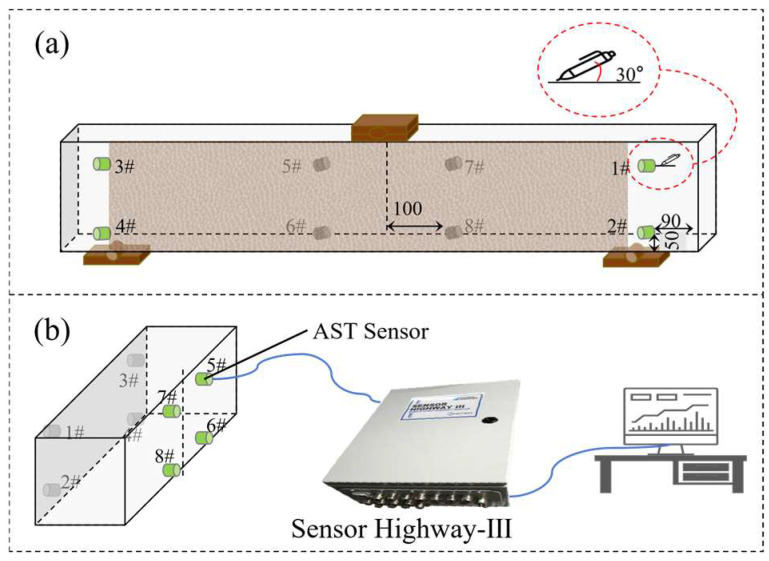
Static test: (**a**) lead breaking test and (**b**) Automatic Sensor Testing.

Before the loading test, a static test was conducted to calibrate the initial wave velocity of the reinforced concrete beam. The static test primarily consisted of pencil-lead break tests. As shown in Figure 3a, a pencil-lead break test was performed at the position of Sensor 1 to determine the initial wave velocity. The pencil-lead break device was set at a 30° angle to the beam surface, and an acoustic emission signal was acquired simultaneously with the lead break. During the load-holding stage, an Automated Sensor Test (AST) was carried out. In this test, as shown in Figure 3b, Sensor 5# was used to generate an excitation signal, which was received by the other sensors. The excitation signal simulated an AE signal to verify the proper functioning of all sensors during the data acquisition process.

**Figure 4:**
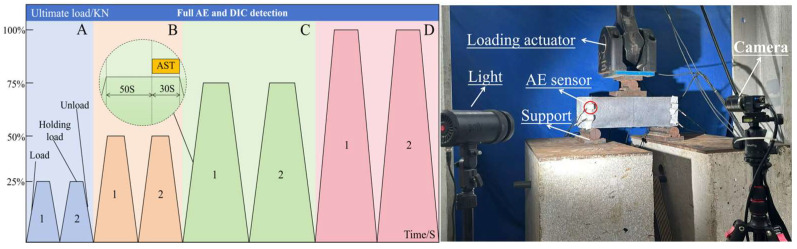
Loading process.

To investigate the manifestation of the Felicity effect in terms of source mechanism and fracture mechanism response, cyclic incremental loading was applied to the reinforced concrete beam. The loading protocol is shown in Figure 4. The loading process was divided into four stages. The four distinct colors correspond to the four stages: A, B, C, and D. Each stage comprised two cycles, and each cycle consisted of three steps: loading, load-holding, and unloading. Each loading step was designated with a specific name. For example, LA1 denotes the loading step of the first cycle in Stage A, while LHA1 indicates the combined loading and holding steps of the first cycle in Stage A. The first cycle of Stage A included the steps: LA1, HA1, and UA1. The load levels for each stage were 25%, 50%, 75%, and 100% of the designed ultimate load-carrying capacity, respectively. To better observe acoustic emission signals associated with crack closure during the holding and unloading states, the loading rate was set to 0.2 kN/s. AE and DIC data were acquired simultaneously during loading.

## 3. Results of Loading Test

### 3.1. Load-Deflection Curves

**Figure 5:**
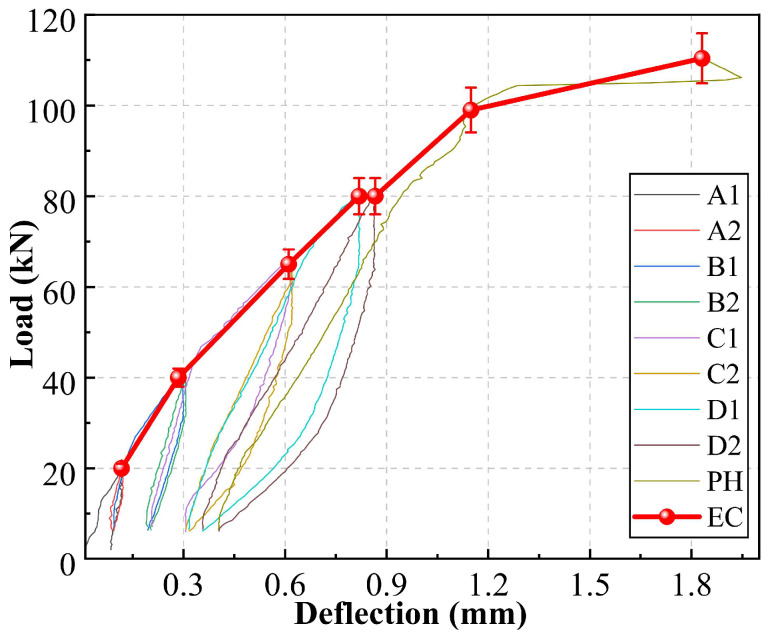
Load-deflection curve.

All mechanical data were obtained from the mean values of three specimens. The coefficient of variation for the key results of the three repeated specimens was less than 5%, indicating good reproducibility of the experiments. To deeply investigate the damage evolution process of reinforced concrete beams under cyclic loading, their macroscopic mechanical responses are first analyzed. Figure 5 shows the load-deflection hysteresis curves of the test beam. In stages A and B, the hysteresis curves appeared narrow and pinched, with the envelope showing a linear ascending trend, and the peak deflections in adjacent cycles basically coincided. This indicates that the specimen was in a linear-elastic working state dominated by elastic deformation, where internal damage mainly consisted of stable micro-crack initiation, with slight stiffness degradation. In stages C and D, the mechanical response changed significantly. The hysteresis loops gradually became fuller. The most notable feature was that, under the same load level, the peak deflection increased cycle by cycle, reflecting continuous damage accumulation. Meanwhile, the slope of the envelope curve slowed noticeably and eventually descended, indicating overall stiffness degradation and the onset of steel yielding. The dominant mechanisms in this stage shifted to stable propagation of macro-cracks and interfacial friction slip. In the final PH stage, after the yield plateau, the bearing capacity began to degrade. This was manifested by a rapid drop in load-carrying capacity after the peak and an abrupt loss of stiffness, marking the unstable failure phase of the specimen. During this stage, mechanisms such as unstable propagation of main cracks and concrete crushing jointly led to the loss of bearing capacity.

### 3.2. Energy Dissipation

**Figure 6:**
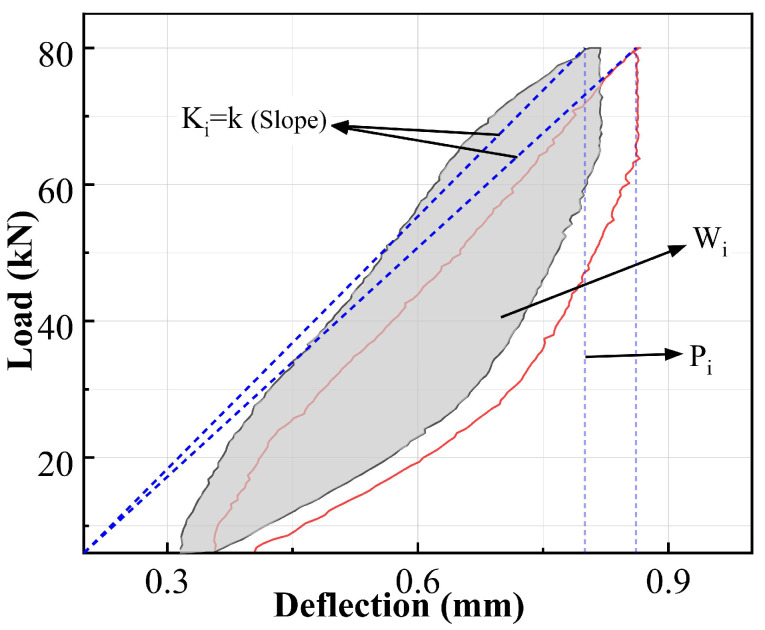
Explanation of the meanings of W_i_ and K_i_. The black line and the red line both represent two cycles within the same period.

To objectively and quantitatively reveal the essence of damage evolution and avoid the uncertainty caused by subjective description, this study extracts two core macroscopic mechanical indicators from the load deflection hysteresis curves for damage stage division: the fracture energy and the secant modulus. Based on the load displacement data, the peak point ($P_{i}$, $\delta_{i}$) of the i-th loading cycle is identified as the fundamental data. The macroscopic fracture energy, $G_{f}$ is first defined as:

(1) $$G_{f}=\sum_{i=1}^{N}W_{i}$$

where $W_{i}$ is the area of the hysteresis loop for the i-th load cycle, representing the energy dissipated by irreversible processes such as friction, plastic deformation, and crack growth during that cycle. Figure 6 shows two cycles from Stage C, where the gray area represents $W_{i}$. The hysteretic loop energy for each cycle was calculated using the trapezoidal numerical integration method applied to the load-deflection data points. The physical meaning of $G_{f}$ lies in characterizing the energy required to create a unit area of damage in the material [41], which is an inherent property of the material’s resistance to fracture.

**Figure 7:**
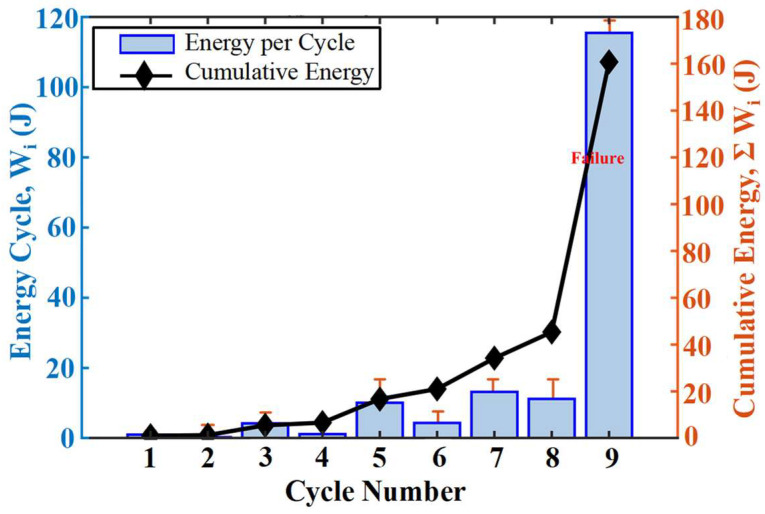
Energy Dissipation Evolution.

The cumulative fracture energy, $\sum W_{i}$ intuitively depicts the accumulation of damage through energy dissipation over the loading history, as shown in Figure 7. The cumulative fracture energy in stages A (cycle 1,2) and B (cycle 3,4) is extremely low, indicating negligible energy dissipation during the damage initiation phase. It increases significantly in stages C (cycle 5,6) and D (cycle 7,8), revealing the dominant role of frictional energy dissipation during stable damage progression. By the PH (cycle 9) stage, the cumulative fracture energy increases exponentially, signifying intense energy release during the failure stage. It is noteworthy that even within repeated loading sub-cycles under a constant load level, a small amount of fracture energy is generated. This phenomenon indicates that stress cycles alone can induce irreversible micro-slip or micro-damage. Relying solely on the response of a single sub-cycle may lead to misjudgment of the damage state. Therefore, a comprehensive assessment based on the full evolution trend is necessary, which will be discussed later.

### 3.3. Stiffness

Secondly, stiffness $K_{i}$ is adopted to characterize the overall average deformation resistance of the member in the i-th cycle. It is defined as the ratio of the load to the deflection at the peak point of that cycle:

(2) $$K_{i}=\frac{P_{i}}{\delta_{i}}$$

**Figure 8:**
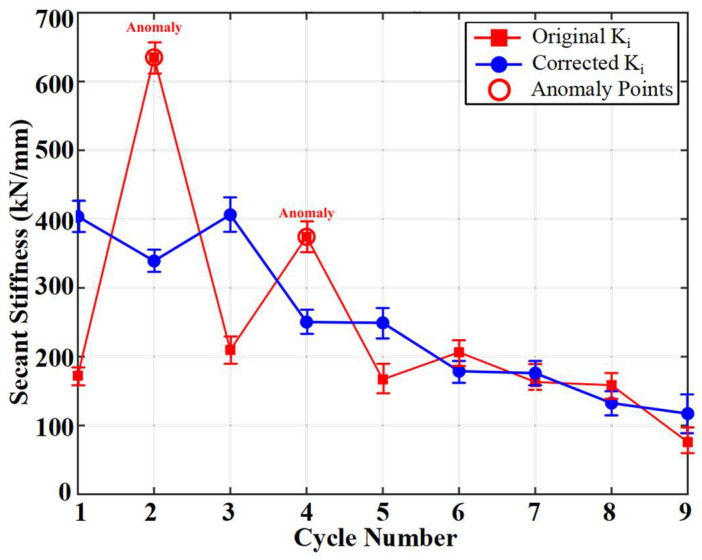
Degradation: Original vs. Corrected.

The decrease in $K_{i}$ directly reflects the overall performance degradation caused by concrete cracking, steel yielding, and interface slip [42], as shown in Figure 6 and Figure 8. $K_{i}$ in cycles 2 and 4 exhibits abnormally high values, which stem from incomplete compaction of the specimen or measurement noise in the initial cycling stage, precisely confirming the slight and uncertain nature of early damage. From cycles 5 to 8, $K_{i}$ decreases significantly and converges to a lower stable plateau, indicating that the member has entered a stable damage development phase characterized by significant stiffness degradation. By cycle 9, K_9_ plummets to 75.91 kN/mm, signaling the final failure of the load-bearing beam.

In the early loading cycles, the contact between the specimen and the supports/loading apparatus was not fully compact. Additionally, at extremely low load and deflection levels, the relative influence of inherent noise in the measurement system was amplified. These factors caused the original secant stiffness $K_{i}$, calculated as the instantaneous ratio of peak load to peak deflection, to exhibit anomalously high values in cycles 2 and 4. These outliers do not represent the material’s true stiffness but rather reflect measurement and systemic errors, which would obscure the judgment of the overall stiffness degradation trend.

To eliminate such random fluctuations and obtain a robust indicator that better reflects the continuous evolution of the structure’s true stiffness, this study applied the moving average method to smooth the original stiffness sequence. Specifically, a simple moving average with a window width of 3 was used. That is, the corrected stiffness $K_{i,\mathrm{corrected}}$ was calculated as the arithmetic mean of the original stiffness values from the cycle in question and its immediate neighboring cycles, $K_{i-1}$, $K_{i}$, and $K_{i+1}$:

(3) $$K_{i,corrected}=\frac{K_{i-1}+K_{i}+K_{i+1}}{N},$$

N represents the actual number of valid data points included in the average (N = 2 at the boundaries). This method effectively suppresses single-point anomalies caused by accidental factors while preserving the overall evolutionary trend of the sequence.

The corrected stiffness sequence is displayed as the Corrected $K_{i}$ curve in Figure 8. After smoothing, the corrected stiffness values remain at a relatively high level with minor fluctuations during cycles 1–4. From cycles 5–8 onwards, the stiffness exhibits a systematic and significant decline, stabilizing at a lower numerical plateau. A sharp drop in stiffness occurs at cycle 9, corresponding to the loss of load-bearing capacity. This smoothed stiffness evolution pattern aligns closely with the damage stages defined based on energy dissipation. Table 3 summarizes the key macroscopic mechanical parameters under the cyclic incremental loading.

### 3.4. Felicity Ratio Analysis

Macro-mechanical parameters objectively characterize the stages and trends of damage evolution from the perspective of overall response, providing an external framework for the research. However, to deeply reveal the mesoscopic fracture mechanisms of damage accumulation, more direct evidence must be sought from within the material. Acoustic emission technology offers a unique perspective for this purpose. Its signals not only reflect the instantaneous activity of damage events but also contain key information regarding damage history and memory effects. Among various acoustic emission parameters, the Felicity Ratio (FR) holds particular value due to its sensitive characterization of damage irreversibility. It is defined as [43]:

(4) $$FR=\frac{\mathrm{The}\;\mathrm{acoustic}\;\mathrm{emission}\;\mathrm{onset}\;\mathrm{load}\;\mathrm{during}\;\mathrm{reloading}}{\mathrm{The}\;\mathrm{historical}\;\mathrm{maximum}\;\mathrm{load}},$$

**Figure 9:**
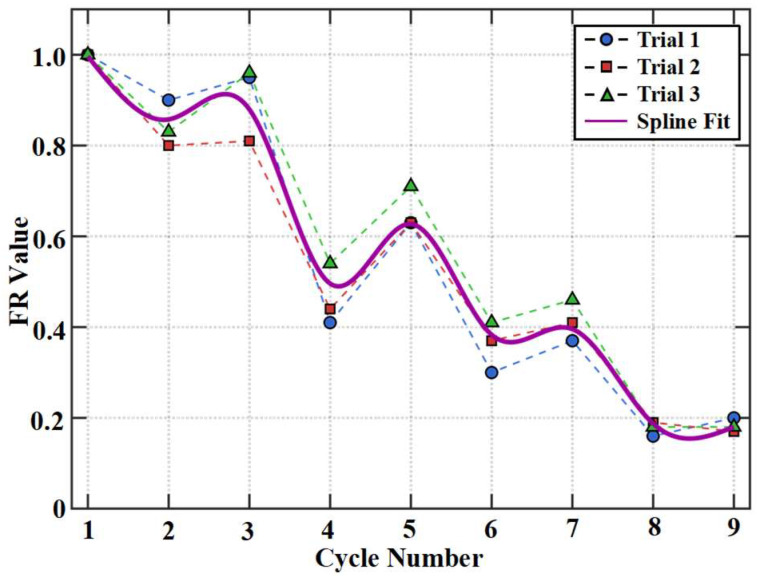
Felicity Ratio.

FR originates from the classic Kaiser effect and Felicity effect. For undamaged or reversibly damaged materials, acoustic emission activity remains silent until the stress exceeds the historical maximum; when the FR value is 1, it represents the Kaiser effect [44]. When irreversible damage accumulates inside the material due to cyclic loading, these defects can be reactivated at lower stress levels during subsequent loading, leading to significant acoustic emission activity occurring earlier, and the FR value consequently decreases, which is the Felicity effect [45]. Therefore, a decline in the FR value from 1.0 serves as the most direct indicator of irreversible damage accumulation, quantifying the extent of material degradation due to damage history. Throughout the entire testing process, the FR value exhibited a significant decreasing trend, as shown in Figure 9. To obtain a continuous curve that smoothly reflects the population statistical behavior of this parameter and has clear physical meaning, we performed spline curve fitting on the discrete FR data points from the three specimens. The FR curve ultimately reported and analyzed in the manuscript is this fitted result, which effectively summarizes the typical evolutionary trend of the FR value with cyclic loading.

During the initial stages A, the FR value approached 1, exhibiting a typical Kaiser effect where acoustic emission activity appeared only when a new load peak was reached. This indicates that the early-stage damage was dominated by reversible elastic deformation or extremely fine microcracks that could fully close, leaving the acoustic emission sources inactive upon unloading. As the number of cycles increased and damage accumulated, the FR value began to decline. After the 4th cycle, it fell into the range of 0.3–0.6, suggesting that microcracks and interfacial damage had formed irreversibly. In subsequent loading cycles, friction or bond slippage along the rough crack surfaces was activated and generated acoustic emission at load levels far below the historical maximum. As final failure approached, the FR value dropped further below 0.3, clearly indicating that damage had severely accumulated and the interior was filled with defects that could be activated under very low stress. Fracture energy showed relatively low dissipation during repeated loading; however, the FR offered a more direct observation of damage occurrence, with its value during repeated loading being even lower than that in the first cycle of incremental loading. The underlying fracture mechanism lies in the fact that microcracks and interfacial debonding generated in previous cycles could not fully heal after unloading, resulting in pre-existing defects.

### 3.5. Failure Modes

**Figure 10:**
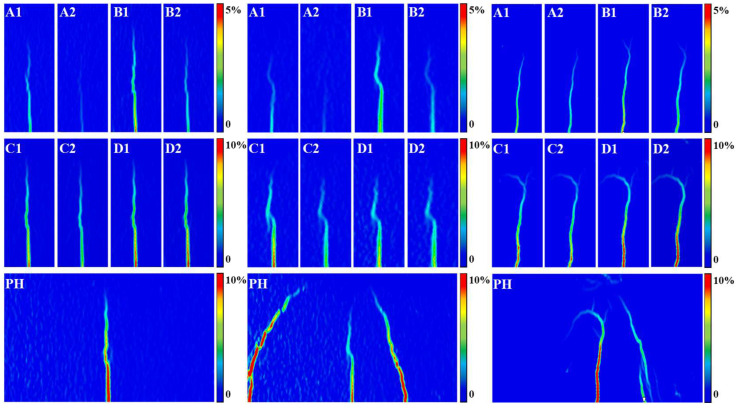
Damage Distribution of the Main Crack Based on DIC Major Tensile Strain.

In this quasi-static cyclic incremental loading test, three typical stages can be identified based on the physical characteristics of damage evolution. The entire failure process can be divided into three distinct phases: the Micro-crack Initiation and Linear Degradation Stage, the Stable Damage Development and Yielding Stage, and the Unstable Failure Stage, as illustrated by the stress-strain contour plot of the reinforced concrete beam failure process shown in Figure 10. The color scale represents the magnitude of principal tensile strain obtained from DIC analysis, highlighting crack paths and damage localization. In the figure, PH denotes the failure stage following the four cyclic stages, which corresponds to the subsequently mentioned Cycle 9 stage.

Stage I: Micro-crack Initiation and Linear Degradation Stage. The stiffness gradually decreased from its initial value, and the Single-Cycle Fracture Energy W_i_ remained at a very low level. Macroscopically, this indicates that damage was dominated by stable micro-cracking within the concrete, and the structural behavior remained nearly linear. Correspondingly, the acoustic emission FR value remained close to 1 throughout this stage, exhibiting a typical Kaiser effect, meaning the material remained silent until the historical maximum stress was reached. From a mesoscopic perspective, this confirms that most of the micro-cracks generated during this stage could close upon unloading. The damage was highly reversible, and no stable permanent defects had yet formed internally.

Stage II: Stable Damage Development and Yielding Stage. The stiffness fluctuated and gradually declined at a relatively low level. The most significant feature was the order-of-magnitude increase in the Single-Cycle Fracture Energy W_i_, which was approximately ten times that of Stage I. Macroscopically, this marked the transition to steel yielding and frictional slip along crack surfaces as the dominant mechanisms [46]. Concurrently, the acoustic emission FR value dropped to approximately 0.3–0.6. The decrease in the FR value reflects the Felicity effect, demonstrating that microcracks and interfacial debonding generated in previous cycles could no longer fully heal, resulting in irreversible pre-existing defects. These defects were reactivated at lower stress levels during subsequent loading, generating new acoustic emission activity. This process reveals the irreversible accumulation of damage from an internal perspective.

Stage III: Unstable Failure Stage. The stiffness undergoes abrupt degradation, and the single-cycle fracture energy Wi skyrockets to 8–10 times that of Stage II. Macroscopically, the main crack enters a state of unstable propagation. At this point, the acoustic emission FR value further drops below 0.3. The extremely low FR value indicates that the material interior is already filled with numerous defects, and even under very low stress levels, severe damage behavior can be triggered. This internal damage serves as an early warning of the impending unstable failure of the component.

## 4. AE Analysis

### 4.1. Frequency-Domain Analysis

The subsequent analysis was based on data from the beam whose acoustic emission signal frequency-domain characteristics were closest to the overall mean. The frequency-domain features of acoustic emission contain key information about fracture mechanisms and can be regarded as the basis for identifying mesoscopic damage. Therefore, this subsection focuses on the frequency-domain characteristics of acoustic emission waveforms, aiming to establish a deterministic correlation between different damage stages and specific fracture mechanisms.

**Figure 11:**
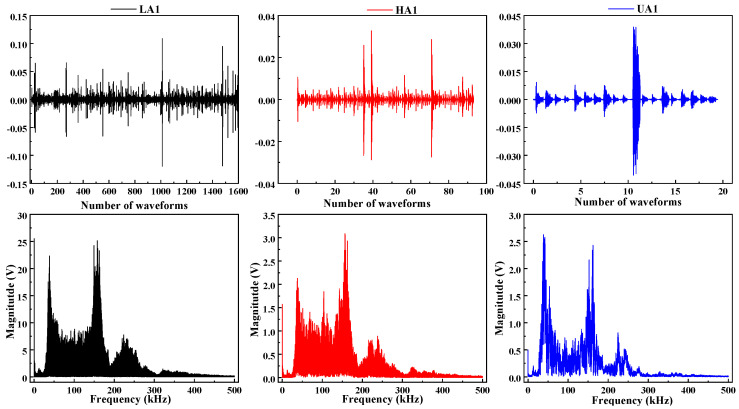
A1 stage full shape of signals.

**Figure 12:**
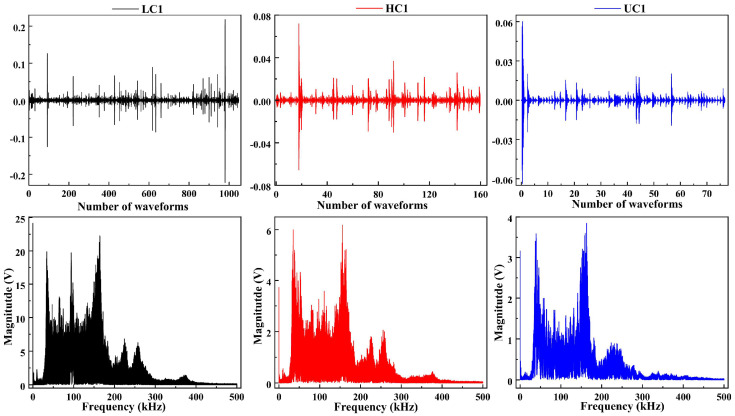
C1 stage full shape of signals.

First, the full shape of signals and their frequency spectra of typical cycles A1 and C1 are compared in Figure 11 and Figure 12. The analysis reveals that although the signal energy is predominantly distributed across three frequency bands, 20–100 kHz, 100–200 kHz, and 200–300 kHz, there are significant differences in their amplitudes and dominant frequencies. The 20–100 kHz band is generally associated with low-frequency vibration of the overall structure, frictional slip at the interface between coarse aggregate and mortar, and stable activity of macroscopic cracks. The 100–200 kHz band primarily corresponds to brittle cracking of the cement matrix at the meso-scale and rapid propagation of micro-cracks. The 200–300 kHz band mainly relates to sudden events such as fracture of fine aggregate.

**Figure 13:**
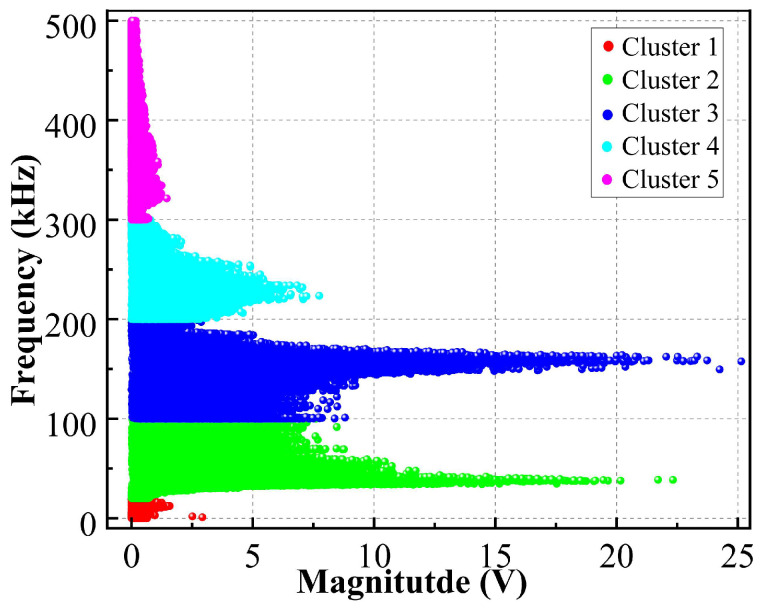
K-means classification results.

To establish an objective basis for frequency band division in this study, a K-means clustering analysis was performed on the frequency-amplitude characteristics of all acquired AE waveforms after Fast Fourier Transform (FFT). The results confirmed that the AE signals naturally clustered into five distinct groups within the frequency spectrum, as shown in Figure 13. These clusters guided the division of the full bandwidth into the following five sub-bands for subsequent quantitative analysis: ultra-low frequency (1–20 kHz), low frequency (20–100 kHz), medium frequency (100–200 kHz), high frequency (200–300 kHz), and ultra-high frequency (300–500 kHz).

**Figure 14:**
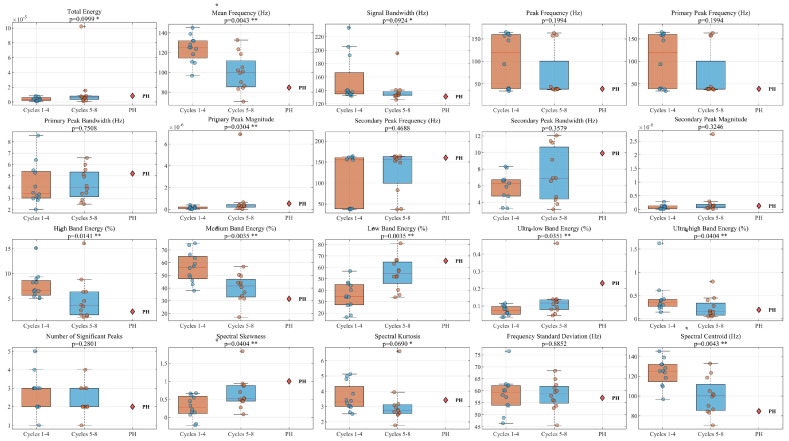
Spectral Differences at Different Damage Stages. In the figure, features with *p* < 0.05 are marked with **, and features with *p* < 0.1, indicating potentially marginally significant differences, are marked with *.

**Figure 15:**
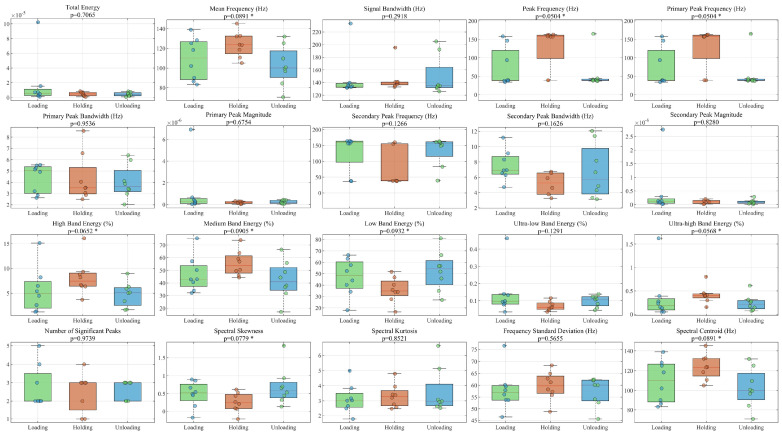
Spectral Differences among Loading, Holding, and Unloading Phases. In the figure, features with *p* < 0.1, indicating potentially marginally significant differences, are marked with *.

Simultaneously, a total of twenty frequency-domain and spectral shape parameters were extracted, including the Mean Frequency, Spectral Skewness, and Spectral Kurtosis. As the AE feature data typically do not meet the assumption of normal distribution, non-parametric tests were employed for significance analysis of between-group differences. The Kruskal-Wallis test was used for comparisons among the loading, holding, and unloading stages, while the Mann-Whitney U test was applied for comparisons among the three macroscopic damage stages. The significance level was set at α = 0.05. In the figures, features with *p* < 0.05 are marked with **, and features with *p* < 0.1, indicating potentially marginally significant differences, are marked with *. The sample sizes of valid AE hit signals for the three damage stages are 3224, 5502, and 1889, respectively. The sample sizes for the loading, holding, and unloading stages are 6359, 1107, and 1261, respectively. The aim was to objectively verify the differences in the acoustic emission signal spectra under different mechanical states or damage levels. The statistical analysis results in Figure 14 clearly show that multiple frequency-domain characteristics of the acoustic emission signals exhibit statistically significant differences among the different damage stages. Figure 15 analyzes the frequency-domain characteristics of the three “loading-holding-unloading” stages, which show differences in the same frequency-domain parameters compared to the different damage stages.

Table 4 presents the *p*-values and ε^2^ values from the significance tests for the 20 feature parameters across the different stages. A *p*-value less than 0.05 indicates a significant difference in the corresponding feature between the groups. The overall sample size for the waveform data is large, resulting in ε^2^ values approaching zero.

The statistical results indicate that, across different damage stages, although multiple frequency-band energy and frequency characteristics show statistical significance at the *p* < 0.05 level, their effect sizes ε^2^ are all below 0.002. This suggests that the actual magnitude of differences in feature values between stages is limited, with considerable overlap in their distributions across groups. Consequently, any single feature is unlikely to reliably distinguish between damage stages.

In contrast, within the same damage stage, the vast majority of features show non-significant *p*-values and extremely low ε^2^ values when comparing the loading, holding, and unloading sub-steps. This confirms that the differences in AE spectral characteristics excited by these three sub-steps are minimal, and their discriminability is significantly lower than the differences observed between distinct damage stages.

Furthermore, to control the potential false positive risk introduced by multiple comparisons, this study applied the Bonferroni correction, setting a strict significance threshold of 0.0025. Even under this corrected standard, three features—mid-band energy, low-band energy, and ultra-low-band energy-retain statistical significance in the damage stage analysis. This indicates that these frequency-band energy characteristics maintain a relatively robust association with damage evolution, supporting their validity as effective discriminative bases in the multi-feature fusion model.

During the Microcrack Initiation Stage, the acoustic emission signals exhibit a higher mean frequency, a greater energy proportion in the high and medium frequency bands, and higher spectral kurtosis, while simultaneously showing the lowest spectral skewness. These characteristics reveal an acoustic emission mechanism dominated by high frequency, sudden emissions, and sharp spectral peaks. The damage at this stage is primarily characterized by a large number of dispersed, fine-scale cement matrix microcracks and interface debonding. Such brittle fracture events occur rapidly and have short characteristic durations, thereby exciting abundant high-frequency components. The signals exhibit a relatively broad frequency band and higher spectral kurtosis, indicating that the signal energy is concentrated at multiple discrete frequency points. This reflects the fracture mechanism of small-scale, randomly distributed microcracking events.

During the Stable Damage Development Stage, stress concentrates and accumulates around the main crack, leading to the formation of macro-cracks. Steel yielding, sliding along macro-crack surfaces, and bond failure become the dominant mechanisms. Correspondingly, the spectral skewness of the acoustic emission signals shows an increasing trend, indicating a change in the asymmetry of the signal spectrum shape and a more noticeable shift of the energy distribution toward lower frequencies. The propagation of macro-cracks and the initial plastic deformation of steel excite more dominant low-frequency components. Furthermore, as acoustic emission waves propagate from the source to the sensor, they must pass through the gradually widening main crack. These cracks act as natural low-frequency filters, causing more significant attenuation and scattering of high-frequency components, which results in an overall downward shift in the frequency content of the received signals.

The frequency-domain characteristics in the Unstable Failure Stage show a trend almost opposite to that of Stage I. They are manifested as the lowest mean frequency, the highest energy proportion in the high-frequency band and the highest spectral kurtosis, coupled with the highest energy proportions in the low-frequency and ultra-low-frequency bands and the highest spectral skewness. This indicates that the signals are characterized by low frequency, continuous emission, broad and gentle spectra with a low-frequency tail. The dominant fracture mechanisms at this stage are the unstable propagation of macro-cracks, the crushing of concrete, and the complete plastic deformation and bond failure of steel reinforcement.

### 4.2. Frequency Bandwidth Energy Evolution

**Figure 16:**
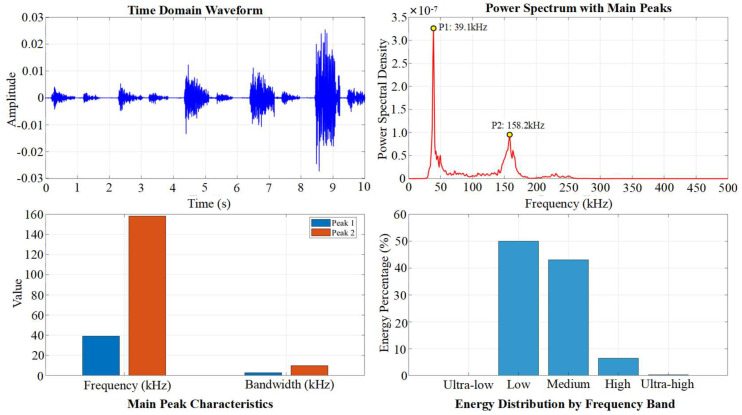
Frequency domain characteristics of cracking signals.

**Figure 17:**
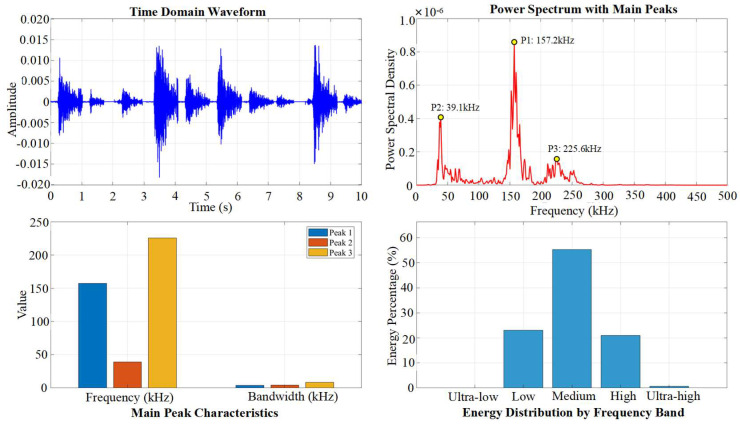
Frequency domain characteristics of the hold signal.

Ten signals were extracted from the loading and holding phases of the Microcrack Initiation Stage, representing cracking and stable holding, respectively, for frequency-band division and analysis of frequency-domain features. The results in Figure 16 and Figure 17 indicate that the differences in frequency-domain signals across stages are mainly reflected in the varying energy proportions of the high-frequency, medium-frequency, and low-frequency bands.

**Figure 18:**
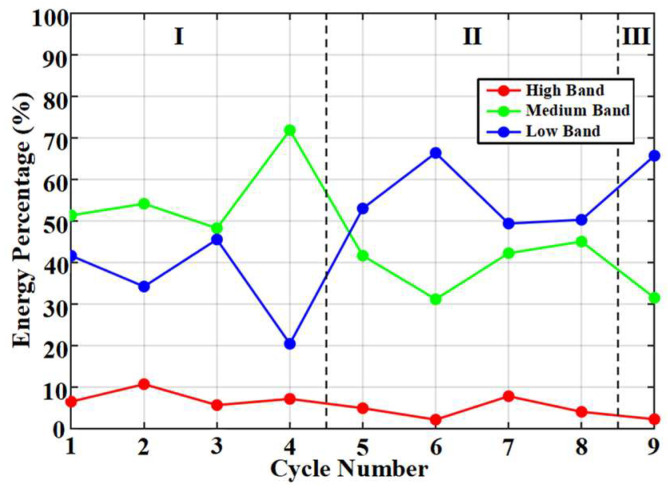
Band energy mechanism transformation. The dotted lines are used to divide different stages of damage.

Accordingly, we divided the energy of these three frequency bands for the nine cycles. To more intuitively reveal this evolutionary pattern, Figure 18 presents the full-process variation trend of the energy proportions for the high-frequency, medium-frequency, and low-frequency bands across the nine complete cycles. This evolutionary trajectory clearly outlines the fundamental transition of the fracture mechanism from being dominated by brittle fracture to being dominated by frictional slip.

Throughout the entire loading process, the proportion of high-frequency band energy remains at a relatively high level in Stage I, which aligns with the mechanism whereby the brittle initiation of microcracks generates a large number of high-frequency signals. Upon entering Stage II and Stage III, the proportion of high-frequency band energy decreases and overall stabilizes at a low and steady plateau. This indicates that as macro-cracks form and propagate, pure brittle fracture events diminish, and their contribution to acoustic emission is overshadowed by other mechanisms.

More distinctive is the “X-shaped” evolutionary relationship between the energy proportions of the medium-frequency and low-frequency bands. In Stage I, the proportion of medium-frequency band energy is significantly higher than that of the low-frequency band. This reflects that although the damage in this stage is dominated by high-frequency brittle fracture, it is already accompanied by a certain scale of microcrack stable propagation or interface micro-slip events with slightly longer durations, which are manifested in the medium-frequency band. Upon entering Stage II, the relationship between the two reverses: the proportion of low-frequency band energy surpasses that of the medium-frequency band and continues to rise, while the proportion of the medium-frequency band begins to decline. This crossover point marks the transition in the dominant fracture mechanism: steel yielding and repeated frictional sliding along crack surfaces become the main sources of energy dissipation, thereby exciting a large number of low-frequency signals. By Stage III, the proportion of low-frequency band energy further increases sharply, significantly widening the gap with the medium-frequency band. This corresponds to the intense frictional and plastic deformation processes during the failure stage, where low-frequency components occupy an absolutely dominant position.

## 5. Development of Damage Model

### 5.1. Feature Selection

To transform the qualitative correlation between the previously revealed acoustic emission mechanisms and fracture mechanisms into a quantifiable and verifiable damage identification tool, this study constructed a damage stage classification model based on multi-feature fusion. By integrating acoustic emission mechanisms with clear physical significance from multiple domains, it comprehensively assesses the damage stage of each cycle, thereby enabling the identification of the structural damage evolution process.

**Figure 19:**
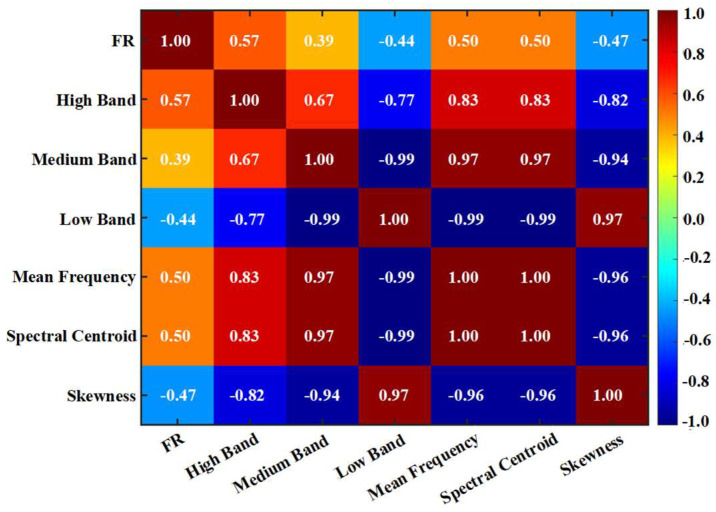
The Pearson correlation coefficients of seven features.

Based on the analysis of acoustic emission mechanisms in Section 3, seven key features were initially selected, including the Felicity Ratio, energy proportion of each frequency band, mean frequency, spectral centroid, spectral skewness, and spectral kurtosis. To avoid high multicollinearity among features and ensure each input variable provides independent damage information, a correlation analysis was performed on the initially selected feature set. Figure 19 revealed strong correlations between the medium-frequency band energy proportion and the spectral centroid, as well as between the low-frequency band energy proportion and the spectral skewness. This indicates significant information overlap among these feature pairs when characterizing damage mechanisms. Therefore, to ensure model efficiency and generalization capability, this study ultimately selected five core features to form the model’s input vector. In fact, the optimal accuracy of the seven-feature model and the five-feature model is the same, hence the five-feature model was chosen to achieve a more concise model. These features not only correspond one-to-one with the specific fracture mechanisms demonstrated in Chapter 3 but also exhibit good statistical independence. They can preserve the damage evolution information to the greatest extent while avoiding issues of multicollinearity.

The model selects five key acoustic emission features as input parameters, each of which is closely associated with specific damage fracture mechanisms:

Felicity Ratio: This parameter is defined as the ratio of the load at which significant acoustic emission activity occurs during repeated loading to the historical maximum load. Its expression is shown in Equation (4).

High-frequency band energy proportion E_high_: This parameter is defined as the percentage of signal energy within the frequency range of 200–300 kHz relative to the total signal energy.

(5) $$E_{\mathrm{high}}=\frac{\int_{200}^{300}PSD\left(f\right)df}{\int_{0}^{f_{\max}}PSD\left(f\right)df},$$

where PSD(f) is the power spectral density function of the signal. As described in Section 3.2, high-frequency components are primarily related to rapid brittle fracture events. Therefore, a high value of E_high_ is an important indicator of the loading stage or the occurrence of new fracture events.

Medium-frequency band energy proportion E_medium_: This parameter is defined as the proportion of signal energy within the frequency range of 100–200 kHz.

(6) $$E_{\mathrm{medium}}=\frac{\int_{100}^{200}PSD\left(f\right)df}{\int_{0}^{f_{\max}}PSD\left(f\right)df},$$

The medium-frequency band energy is associated with multiple mechanisms, including crack propagation with certain durations, coarse aggregate fracture, and crack closure friction during unloading. Its evolution reflects the transition of damage from microcracking to macro-cracking and frictional mechanisms.

Low-frequency band energy proportion E_low_: This parameter is defined as the proportion of signal energy within the frequency range of 20–100 kHz.

(7) $$E_{\mathrm{low}}=\frac{\int_{20}^{100}PSD\left(f\right)df}{\int_{0}^{f_{\max}}PSD\left(f\right)df},$$

Low-frequency components are primarily excited by sustained structural damage behaviors, such as frictional sliding along crack surfaces, bond-slip between steel reinforcement and concrete, and stress redistribution. Therefore, a significant increase in E_low_ serves as an indicator of the holding phase or the dominance of frictional mechanisms.

Mean frequency f_mean_: This parameter is the weighted average frequency of the signal power spectrum, serving as a measure of the center of the signal’s frequency spectrum distribution.

(8) $$f_{\mathrm{mean}}=\frac{\int_{0}^{f_{\max}}f\cdot PSD\left(f\right)df}{\int_{0}^{f_{\max}}PSD\left(f\right)df},$$

The f_mean_ comprehensively reflects the frequency-domain characteristics of the signal. It can effectively capture the overall spectral shift caused by changes in the damage mechanism.

### 5.2. FR-Freq Damage Model Development

Based on the fracture energy and stiffness, the experimental data were explicitly classified into three reference damage stages, serving as the ground truth labels for model training and validation. Statistical analysis of the feature values for each stage was conducted to determine the thresholds for distinguishing between different damage stages. These thresholds are not arbitrarily set but are based on the acoustic emission mechanisms revealed in Section 3 and their specific manifestations during damage evolution:

In Stage I, it is typically close to 1, reflecting the initial Kaiser effect. As irreversible damage accumulates, it decreases to within the range of 0.3–0.6 in Stage II, marking the stable accumulation of irreversible damage. In Stage III, immediately prior to failure, it further drops below 0.3, indicating severe internal defects and the near failure of the memory effect.

Stage I is characterized by relatively high activity in E_high_ and E_medium_. In Stage II, E_low_ begins to increase. Stage III is marked by E_low_ becoming significantly dominant, while E_high_ drops to its lowest level.

It shows a decreasing trend from Stage I to Stage III, consistent with the transition of the fracture mechanism from high-frequency brittle fracture to low-frequency frictional slip.

Based on this, for each feature F_k_, where k=1, 2, …, 5, two thresholds T_k,1-2_ and T_k,2-3_ are set to map the feature values to stage predictions S_k_:

(9) $$S_{K}=\left\{\begin{array}{l} 1(stageI),if\;F_{k}>T_{k,1-2}\\ 2(stageII),if\;F_{k,2-3}<F_{k}\leq T_{k,1-2}\\ 3(stageIII),if\;F_{k}\leq T_{k,2-3} \end{array}\right.\;,$$

Taking the FR value as an example, its decision rule is:

(10) $$S_{FR}=\left\{\begin{array}{l} 1,if\;FR>0.6\\ 2,if\;0.29<FR\leq 0.6\\ 3,if\;FR\leq 0.29 \end{array}\right.,$$

Similarly, threshold-based decision rules are established for the high-frequency band energy proportion, medium-frequency band energy proportion, low-frequency band energy proportion, and mean frequency, respectively. For the i-th cycle, after the five features independently determine their stage predictions, a set of stage prediction values is obtained. The thresholds for other parameters are listed in Table 5.

The final damage stage prediction ${\hat{D}}^{\left(i\right)}$ is determined using a majority voting method for fusion decision making:

(11) $${\hat{D}}^{\left(i\right)}=F(S_{FR}^{\left(i\right)},S_{E_{high}}^{\left(i\right)},S_{E_{medium}}^{\left(i\right)},S_{E_{low}}^{\left(i\right)},S_{f_{mean}}^{\left(i\right)}),$$

After obtaining the independent stage predictions from the five features, a majority voting method is employed for the final decision. That is, the stage that appears most frequently is taken as the final predicted damage stage for that cycle. In case of a tie, the more conservative stage (i.e., the stage indicating more severe damage) is favored. The reference labels for the damage stages were determined manually based on a comprehensive assessment of macroscopic mechanical trends; the model’s automatic identification of unknown cycles is achieved through the aforementioned multi-feature fusion decision function. The F(S) function ultimately returns the mode in the array. This fusion strategy essentially assigns equal weight to each feature, reducing the risk of misjudgment that a single feature might introduce due to noise or random events through collective decision-making, thereby enhancing the model’s robustness.

**Figure 20:**
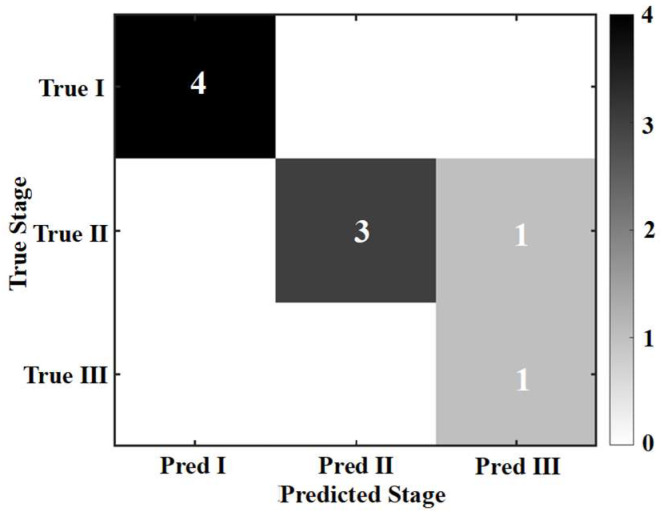
Confusion Matrix.

**Figure 21:**
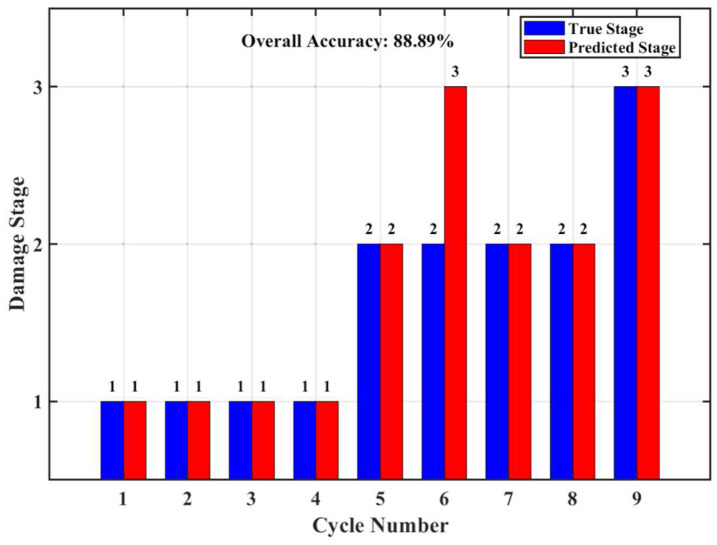
Accuracy of single cycle recognition.

When applying the above model to the experimental data, its overall classification accuracy reached 88.89%, indicating that the FR-Freq damage model can identify the macroscopic damage stages of reinforced concrete beams under cyclic loading with high reliability. This excellent performance validates the core argument proposed in Chapter 3—different damage stages and their dominant meso-scale fracture mechanisms do indeed excite acoustic emission characteristic mechanisms that are statistically distinguishable. The confusion matrix (Figure 20) shows that the identification accuracy for both Stage I and Stage III is 100%, demonstrating that the model’s diagnosis of the initial damage state and the final failure state is extremely reliable. Among the 4 cycles in Stage II, 3 were correctly identified, with only Cycle 6 being misclassified as Stage III, as shown in Figure 21.

**Figure 22:**
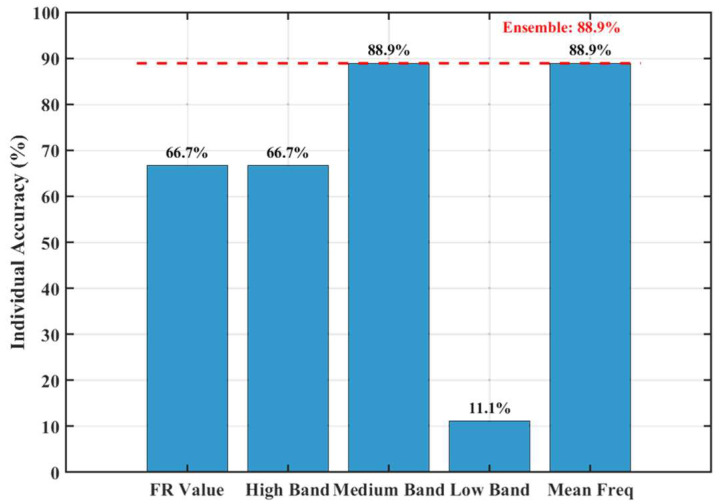
The accuracy rate of the five features’ recognition.

To investigate the root cause of this misclassification, we analyzed the independent identification accuracy of each feature (Figure 22). The results show that the accuracy rates for the four features FR, E_high_, E_medium_, and f_mean_ were 66.7%, 66.7%, 88.9%, and 88.9%, respectively, while the accuracy for E_low_ was only 11.1%. This indicates that the low-frequency band energy proportion is a highly unstable feature in this experimental dataset, prone to misjudgment. A deeper analysis of the specific feature values for Cycle 6 revealed that its E_low_ was abnormally high, reaching levels typical of Stage III, which triggered the model’s misclassification rule. The fracture mechanism behind this phenomenon lies in the fact that Cycle 6 was in the middle of the stable damage development stage. At this point, macro-cracks had formed but their propagation might have been in an intermittent phase. Frictional activity along the crack surfaces might have experienced an abnormal, intense low-frequency friction event due to factors like aggregate interlock or local stress redistribution. This caused the E_low_ feature to transiently exhibit an intensity similar to that of the final failure stage, influencing the final decision in the majority voting process.

This misclassification does not undermine the model’s reliability; instead, it conversely validates the physical logic underlying the model’s construction. It reveals that during the stable damage development and yielding stages, fracture mechanisms can exhibit interweaving and fluctuations. A single feature, especially E_low_, which is sensitive to friction, may temporarily deviate from the actual outcome due to interference from local events. However, the overall model integrating five features still achieved high accuracy of 88.89%. By synthesizing multiple complementary acoustic emission mechanisms, the FR-Freq damage model can effectively filter out local disturbances and capture the dominant trend of damage evolution. FR quantifies the irreversibility of damage, E_high_ captures the activity of brittle fracture, E_medium_ and E_low_ together characterize the evolution of frictional mechanisms, and f_mean_ reflects the overall spectral shift. Their integrated representation enables a mechanistic interpretation of the damage progression.

## 6. Conclusions and Prospects

Through cyclic incremental loading tests on reinforced concrete beams combined with acoustic emission and digital image correlation monitoring, this study systematically revealed the intrinsic correlation between fracture mechanisms and acoustic emission mechanisms, and constructed an FR-Freq damage model based on acoustic emission mechanisms. The main conclusions are as follows:

(1) The results of damage stage classification based on mechanical response are highly consistent with those based on acoustic emission response. The fracture energy extracted from mechanical data established based on Ki demonstrate, from the perspective of fracture mechanisms, the reliability of the acoustic emission time-domain parameter FR for damage stage classification. Their combination allows the damage stages to be clearly divided into: the Microcrack Initiation and Linear Degradation Stage, the Stable Damage Development and Yielding Stage, and the Unstable Failure Stage.

(2) Damage accumulation and irreversible processes can be clearly characterized by acoustic emission activity. During the damage process, stages of repeated loading cycles that elicit weak responses in fracture energy can be distinctly reflected in the FR. This indicates that FR can achieve quantitative characterization of the dynamic process of damage accumulation and respond to damage at an earlier stage

(3) The dominant mesoscopic fracture mechanisms in different stages of cyclic loading excite acoustic emission spectral mechanisms with significant differences. Specifically, the initial brittle fracture of the concrete matrix or aggregate manifests as burst-type signals with prominent high-frequency energy components and high mean frequency. Stable frictional slip along existing crack surfaces excites continuous-type signals dominated by low-frequency energy. The crack closure process during the unloading stage is accompanied by slight friction signals exhibiting mixed mid-to-low frequency characteristics.

(4) The FR-Freq damage model, constructed by integrating multi-domain acoustic emission features, is capable of tracking and verifying the damage evolution process with high accuracy. The proposed model, which incorporates five features, the FR value, high-, medium-, and low-frequency band energy proportions, and the mean frequency, can accurately classify the damage stages, achieving an accuracy of 88.89%.

This study primarily focuses on the macro and meso scales. Future work could integrate microstructural characterization techniques such as scanning electron microscopy (SEM) or computed tomography (CT) to more precisely reveal the relationship between the initiation of initial microcracks in concrete and specific acoustic emission frequency-domain characteristics. The FR-Freq model developed in this study has demonstrated high accuracy in identifying damage stages under low-cycle, incremental cyclic loading. Under high-cycle fatigue with much lower stress amplitudes, AE signal characteristics may differ, and the accumulation of damage might be more gradual. The effectiveness and the need for possible recalibration of the feature thresholds proposed in this model under true fatigue loading conditions warrant further investigation. Nevertheless, this study establishes a valuable correlation between AE features and mesoscopic fracture mechanisms, offering a new perspective for fatigue damage monitoring rooted in physical mechanism recognition rather than purely phenomenological data fitting.

## Figures and Tables

**Table 1:** The concrete mix proportions.

Material	Water (kg)	Cement (kg)	Sand (kg)	Gravel (kg)
Mix Proportion	232.00	464.00	655.00	1069.00

**Table 2:** The reinforcing steel materials.

Diameter (mm)	Nominal Area (mm^2^)	Tensile Strength f_y_ (MPa)	Ultimate Tensile Strength (MPa)	Elasticity Modulus E_s_ (N/mm^2^)	Yield Strain (×10^−6^)	Percentage Elongation
8	50.3	450	618	2.13	2220	--
10	78.5	445	615	2.18	2171	25.2

**Table 3:** The mechanical parameters.

Cycle Number	Secant Stiffness K_i_ (kN/mm) *	Corrected K_i_ (kN/mm)	Single Cycle Fracture Energy W_i_ (J) *	Peak Load P_max_ (kN)	Peak Deflection δ_max_ * (mm)
1	172.56	403.74	0.99	20	0.116
2	634.92	339.08	0.27	20	0.119
3	209.75	406.28	4.21	40	0.285
4	374.18	250.3	1.13	40	0.301
5	166.97	249.19	10.08	65	0.597
6	206.41	178.85	4.32	65	0.621
7	163.17	176.03	13.16	80	0.806
8	158.51	132.53	11.16	80	0.862
9	75.91	117.21	115.44	109.8 *	1.849

* The data represent the average value of the three specimens.

**Table 4:** *p* and ε^2^.

Feature	Different Damage Stages *p*	Different Damage Stages ε^2^	Loading, Holding, and Unloading Phases *p*	Loading, Holding, and Unloading Phases ε^2^
Total Energy	0.0999	0.0004	0.7065	0.0001
Mean Frequency	0.0043	0.0018	0.0891	0.0003
Signal Bandwidth	0.0924	0.0005	0.2918	0.0001
Peak Frequency	0.1994	0	0.0504	0
Primary Peak Frequency	0.1994	0	0.0504	0
Primary Peak Bandwidth	0.7508	0	0.9536	0
Primary Peak Magnitude	0.0304	0	0.6754	0
Secondary Peak Frequency	0.4688	0	0.1266	0
Secondary Peak Bandwidth	0.3579	0	0.1626	0
Secondary Peak Magnitude	0.3246	0	0.8280	0
High Band Energy	0.0141	0.0012	0.0652	0.0004
Medium Band Energy	0.0035	0.0019	0.0905	0.0003
Low Band Energy	0.0035	0.0019	0.0932	0.0002
Ultra-low Band Energy	0.0035	0.0018	0.1291	0.0003
Ultra-high Band Energy	0.0404	0.0008	0.0568	0.0004
Number of Significant Peaks	0.2801	0	0.9739	0
Spectral Skewness	0.0404	0.0008	0.0779	0.0003
Spectral Kurtosis	0.069	0.0006	0.8521	0.0001
Frequency Standard	0.8852	0	0.5655	0
Spectral Centroid	0.0043	0.0018	0.0891	0.0003

**Table 5:** Parameter threshold.

Feature	FR	E_high_	E_medium_	E_low_	f_mean_
Threshold 1	0.29	3.6	35.8	60.2	92.2
Threshold 2	0.6	6.2	48.2	45.1	111.8

## Data Availability

The original contributions presented in this study are included in the article. Further inquiries can be directed to the corresponding author.

## Abbreviations

The following abbreviations are used in this manuscript:

RC	Reinforced concrete
AE	acoustic emission
DIC	Digital image correlation
FR	Felicity Ratio
